# Synergistic Activation of Dopamine D1 and TrkB Receptors Mediate Gain Control of Synaptic Plasticity in the Basolateral Amygdala

**DOI:** 10.1371/journal.pone.0026065

**Published:** 2011-10-14

**Authors:** Chenchen Li, Joanna Dabrowska, Rimi Hazra, Donald G. Rainnie

**Affiliations:** 1 Behavioral Neuroscience and Psychiatric Disorders, Yerkes National Primate Research Center, Atlanta, Georgia, United States of America; 2 Department of Psychiatry, Emory University School of Medicine, Atlanta, Georgia, United States of America; Hokkaido University, Japan

## Abstract

Fear memory formation is thought to require dopamine, brain-derived neurotrophic factor (BDNF) and zinc release in the basolateral amygdala (BLA), as well as the induction of long term potentiation (LTP) in BLA principal neurons. However, no study to date has shown any relationship between these processes in the BLA. Here, we have used *in vitro* whole-cell patch clamp recording from BLA principal neurons to investigate how dopamine, BDNF, and zinc release may interact to modulate the LTP induction in the BLA. LTP was induced by either theta burst stimulation (TBS) protocol or spaced 5 times high frequency stimulation (5xHFS). Significantly, both TBS and 5xHFS induced LTP was fully blocked by the dopamine D1 receptor antagonist, SCH23390. LTP induction was also blocked by the BDNF scavenger, TrkB-FC, the zinc chelator, DETC, as well as by an inhibitor of matrix metalloproteinases (MMPs), gallardin. Conversely, prior application of the dopamine reuptake inhibitor, GBR12783, or the D1 receptor agonist, SKF39393, induced robust and stable LTP in response to a sub-threshold HFS protocol (2xHFS), which does not normally induce LTP. Similarly, prior activation of TrkB receptors with either a TrkB receptor agonist, or BDNF, also reduced the threshold for LTP-induction, an effect that was blocked by the MEK inhibitor, but not by zinc chelation. Intriguingly, the TrkB receptor agonist-induced reduction of LTP threshold was fully blocked by prior application of SCH23390, and the reduction of LTP threshold induced by GBR12783 was blocked by prior application of TrkB-FC. Together, our results suggest a cellular mechanism whereby the threshold for LTP induction in BLA principal neurons is critically dependent on the level of dopamine in the extracellular milieu and the synergistic activation of postsynaptic D1 and TrkB receptors. Moreover, activation of TrkB receptors appears to be dependent on concurrent release of zinc and activation of MMPs.

## Introduction

Evidence from behavioral and electrophysiological studies indicates that the induction of long term potentiation (LTP) in principal neurons of the basolateral amygdala (BLA) may underlie the acquisition and consolidation of fear memories [1], [2]. Significantly, fear memory formation is critically dependent on the activation of dopaminergic afferents to the amygdala. Total dopamine depletion prevents fear memory formation, an effect that can be reversed by selective restoration of dopamine release in the pathway from the ventral tegmentum to the BLA [3]. Moreover, the D1 family of dopamine receptors bi-directionally modulates fear memory formation, with activation facilitating and inhibition attenuating recall [4], [5]. Consistent with this observation, activation of the amygdala in response to fearful faces is dependent on D1 but not D2 receptor occupancy [6]. We have shown that D1 receptors are found in close association with NMDA receptors in the spines of BLA principal neurons [7], where they function to modulate excitatory synaptic transmission [8]. Hence, D1 receptors appear to be optimally positioned to regulate the induction and expression of LTP in afferent inputs to the BLA. Consistent with this hypothesis, the D1 receptor antagonist, SCH23390, blocks low-frequency stimulation-induced LTP in cortical inputs to the lateral amygdala [9], and D1 receptor activation enhances both the duration and the magnitude of LTP elsewhere in the brain [10].

Similarly, brain-derived neurotrophic factor (BDNF) has been implicated in many forms of synaptic plasticity associated with fear memory formation, including LTP [11], [12]. High levels of BDNF and its cognate receptor, tyrosine kinase receptor B (TrkB), are found in the BLA [13], [14], and recent studies have shown that TrkB activation in the BLA is necessary for the acquisition and consolidation of fear memories [14], [15]. Consistent with these data, a recent study has shown that the non-peptide TrkB receptor agonist, 7, 8-dihydroxyflavone, enhanced both the acquisition of fear and its extinction [16]. Moreover, point mutations of the two main phosphorylation docking sites on the TrkB receptor have been shown to modulate the both acquisition and consolidation of fear learning and amygdala synaptic plasticity [17]. Together these data suggest that BDNF and dopamine may play similar roles in BLA-dependent fear learning and memory. Intriguingly, in striatal neurons D1 receptor stimulation can trans-activate TrkB receptors [18], and in the hippocampus dopamine-mediated persistence of long-term memory (LTM) is reported to be mediated by BDNF [19], further suggesting that a synergistic interaction between the dopamine and BDNF systems could play a similar role in BLA-dependent fear memory formation. While synaptic plasticity underlying fear memory formation is assumed to occur in BLA principal neurons, to date no studies have directly addressed the role of D1 receptor activation on LTP in the BLA, or the role of TrkB receptor activation on LTP, specifically in this cell population. The present whole-cell patch clamp recording study was designed to address these knowledge gaps and determine whether these two systems act independently or synergistically to regulate synaptic plasticity in principal neurons of the BLA.

## Results

### LTP induction in BLA principal neurons

**Figure 1:**
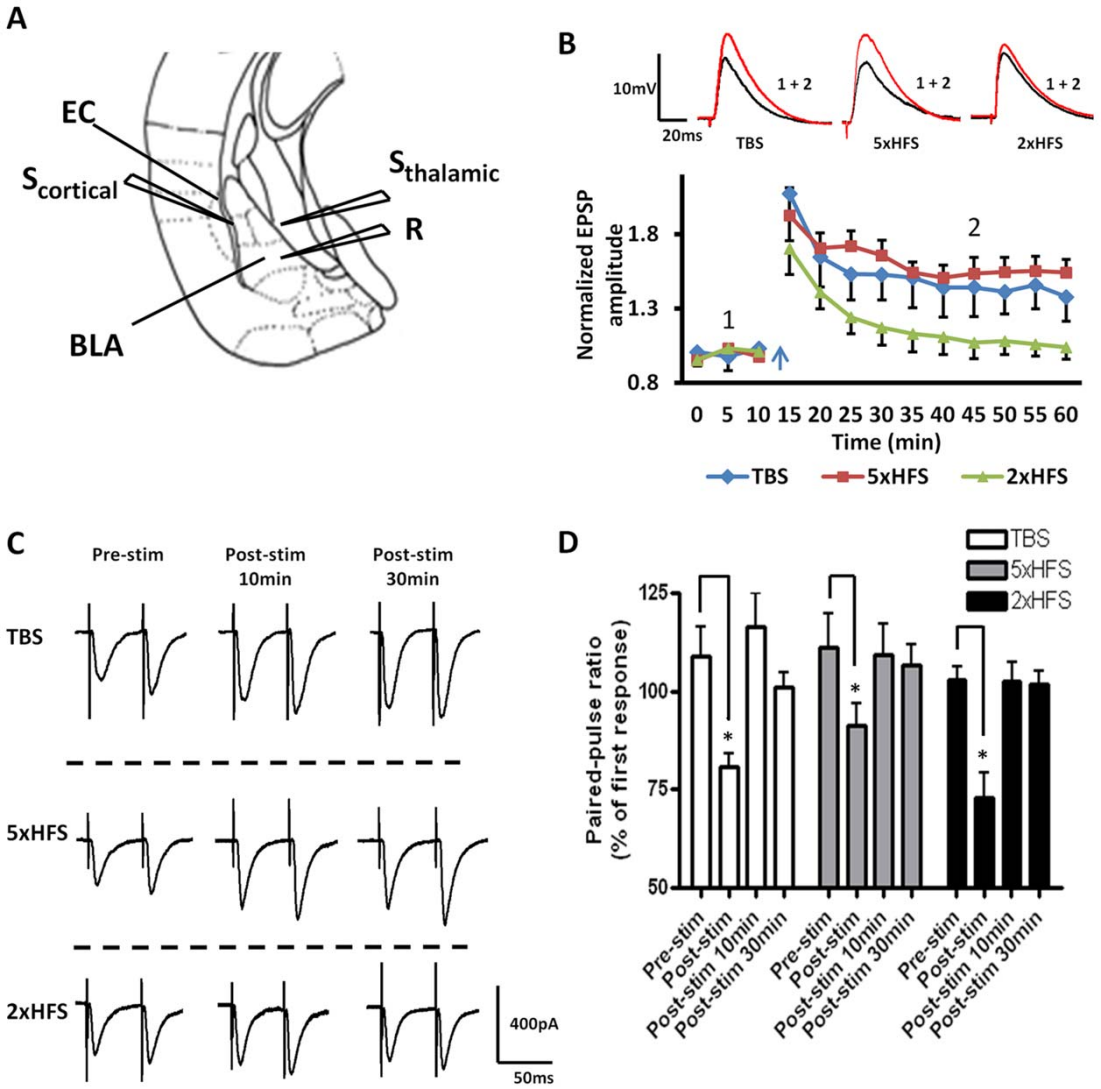
Stimulus dependent induction of LTP in BLA principal neurons. **A**) A schematic illustrating the position of the stimulation (S_cortical_ and S_thalamic_) and recording (R) electrodes. EC, external capsule. **B**) Stimulation of the EC elicits long term synaptic plasticity in BLA afferents following TBS (n = 6, blue diamonds) and 5×100 Hz (n = 5, red squares), but not 2×100 Hz (n = 8, green triangles). **C**) Raw data show the effects of different stimulation protocols on the PPR of EPSCs. EPSC amplitude was measured using paired stimuli pulses separated by 50 ms, before, immediately after, and 10 min and 30 min post stimulation. **D**) A bar chart showing the group data for the effects of stimulation protocol on PPR. Note the large decrease (P <0.05) immediately post-stim, indicating the presynaptic component of post-tetanic-potentiation. * P<0.05. Error bars indicate S.E.M.

Most *in vitro* studies that have examined the cellular mechanisms underlying LTP formation in the BLA have used bath application of GABA_A_ receptor antagonists to block fast inhibitory synaptic transmission and isolate evoked EPSPs. However, we have shown that global reduction of GABA_A_ receptor-mediated synaptic transmission in the BLA can result in profound changes in excitatory drive that would confound the interpretation of any subsequent analysis of LTP [20]. To circumvent this potential confound, we evoked monosynaptic EPSPs (eEPSPs) in BLA principal neurons following stimulation of the external capsule (EC, **Figure 1A**) by including picrotoxin (50 µM) in the patch pipette to block GABA_A_ receptor-mediated synaptic transmission only in the principal neuron under study.

Multi-electrode recording studies in freely moving animals have shown that coherent theta frequency oscillations occur between the BLA and mPFC [21] and between the BLA and hippocampus [22] during fear memory acquisition and recall. Hence, we reasoned that theta frequency modulation of synaptic transmission onto BLA principal neurons may play a significant role in fear memory formation. Consequently, we first examined the effects of theta-burst stimulation of the EC (TBS, see Methods) on the amplitude of eEPSPs in BLA principal neurons. Here, TBS consisted of 40ms bursts of high frequency stimulation (HFS) applied to the EC at 5Hz for 5 seconds. As illustrated in **Figure 1B**, TBS of the EC induced a LTP of the eEPSP that persisted for >45 minutes (blue diamonds). When measured 30 min following TBS, the peak EPSP amplitude was significantly greater than that of the baseline eEPSP amplitude (144±19%, n = 6, 4 animals, P<0.001). Two-way ANOVA revealed no significant effect of treatment at any time point for either the access resistance or the membrane input resistance (F (10, 110) = 0.57, P = 0.98). We next examined the effects of spaced 5xHFS (5x100Hz/1s trains at 20s intervals) of the EC on LTP induction in BLA principal neurons. In the hippocampus, TBS and 5xHFS induce LTP by activating the same cAMP second messenger cascade [23], [24], [25], but the response to 5xHFS was reported to be more robust. Like TBS, 5xHFS of the EC induced a robust LTP of the eEPSP in BLA principal neurons (155±3% of baseline, n = 5, 3 animals, P<0.001, **Figure 1B**, red squares). Two-way ANOVA revealed no effect at any time point of treatment for either the access resistance or the membrane input resistance (F (10, 110) = 0.24, P = 0.99). Thus, both TBS and 5xHFS induced a significant LTP of the eEPSP amplitude in BLA principal neurons.

We next determined whether an abbreviated HFS protocol (2×100Hz/1s trains at 20s intervals; 2xHFS) could induce LTP in BLA principal neurons. In control ACSF, 2xHFS caused a brief post-tetanic potentiation of the ePSP (PTP; 141±11% of baseline) that rapidly decayed back to baseline after 20 min (113±12% of baseline, n = 8, 5 animals; **Figure 1B**, green triangles). Hence, 2xHFS was used in subsequent experiments as a sub-threshold protocol to examine the effects of drug treatment on the sensitivity of BLA principal neurons to LTP induction. Two-way ANOVA revealed no significant effect of treatment at any time point for either the access resistance or the membrane input resistance (F (9, 100) = 0.26, P = 0.9825). Moreover, because LTP induction by TBS was more variable than the response to 5xHFS in most subsequent experiments we used 5xHFS to study the mechanisms underlying LTP induction in BLA principal neurons.

To examine any potential effects of treatment on presynaptic release we also examined the paired-pulse ratio of eEPSCs (PPR, see Methods), a measure of short-term presynaptic plasticity, before, immediately after, as well as 10 min and 30 min after each of the LTP induction protocols (TBS, 5xHFS and 2xHFS). Typical examples of the paired-pulse response before and after each of the stimulation protocols are illustrated in **Figure 1C**. Group data is illustrated in **Figure 1D**. As expected all three stimulation protocols elicited a significant reduction in PPR during the PTP response immediately following the stimulation (P<0.05, n = 6, 4 animals), whereas the PPR was not significantly different from control values 10 or 30 min after the stimulation. These data suggest that the LTP induced by either TBS or 5xHFS was most likely postsynaptic and not presynaptic in origin.

**Figure 2:**
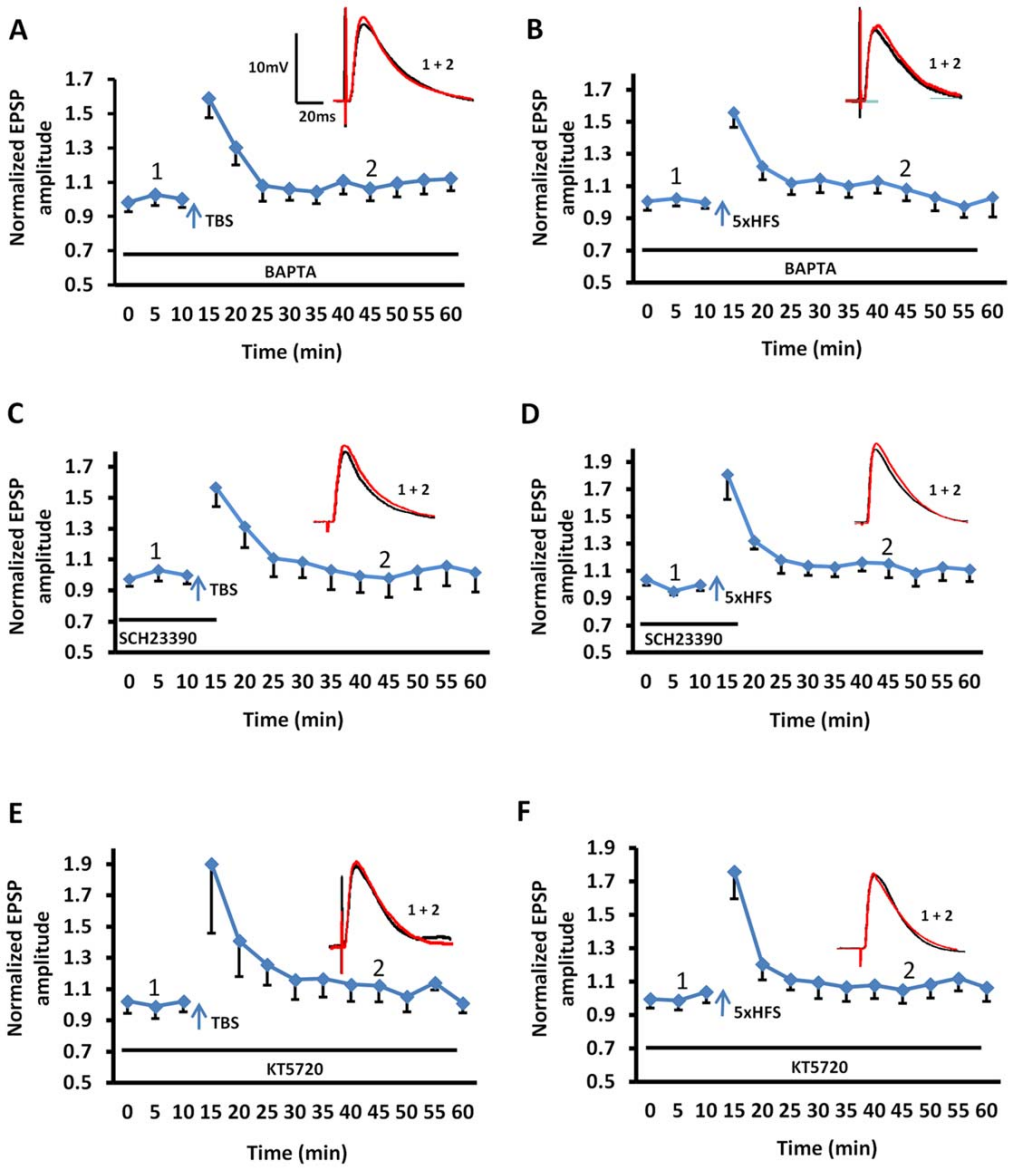
Calcium influx, activation of D1 receptors, and induction of the cAMP-PKA cascade are all required for the induction and maintenance of LTP in BLA principal neurons. **A, B**) Inclusion of the calcium chelator, BAPTA (10 mM) in the patch pipette, fully blocked the induction of LTP by either TBS (n = 6) or 5xHFS (n = 5). **C, D**) Endogenous dopamine was released during TBS and 5xHFS and was necessary for the induction of LTP in BLA principal neurons. Incubation with SCH23390 (10 µM) before and during TBS (n = 6) or 5xHFS (n = 6) completely abolished the induction of LTP. **E, F**) The induction LTP by either TBS or 5xHFS was dependent on activation of the cAMP-PKA signaling cascade. Intracellular application of PKA inhibitor KT5720 (100 nM) fully blocked TBS-induced LTP (n = 5), as well as 5xHFS-induced LTP (n = 5).

Consistent with previous reports [26], inclusion of the calcium chelator, BAPTA (10 mM) in the patch pipette, fully blocked the induction of LTP by either TBS (106±7% of baseline, n = 6, 4 animals; **Figure 2A**) or 5xHFS (104±6% of baseline, n = 5, 3 animals; **Figure 2B**), further supporting a postsynaptic origin for this response. Moreover, intracellular application of BAPTA caused no enduring change in input resistance at any time point measured (F (16, 136) = 1275, P = 1.27, Two-way ANOVA).

Finally, previous studies had reported that LTP induction in the lateral amygdala was pathway specific [27], [28]. Hence, in a parallel study we examined the response of BLA principal neurons to 5xHFS of the thalamic input. In contrast to the response to 5xHFS of the EC, stimulation of the thalamic input induced only a transient increase in the eEPSP amplitude in BLA principal neurons that was not significantly different from baseline when measured 30 min after 5xHFS (119±8% of baseline amplitude, n = 6, 4 animals, P<0.05; data not shown).

### Dopamine-dependency of LTP induction in BLA principal neurons

In vivo studies have shown that fear learning is dependent on dopamine release in the BLA, and subsequent activation of dopamine D1 receptors [3]. Consequently, we next examined the role of D1 receptor activation on the induction of LTP in BLA. Here, slices were pretreated with the D1 receptor antagonist, SCH23390 (10 µM), for 10 min prior to, and during TBS. Bath application of SCH23390 caused a significant attenuation of TBS-induced LTP in BLA neurons (98±12% of baseline, n = 6, 4 animals, **Figure 2C**). These data not only suggested that TBS induced the release of endogenous dopamine, but also that subsequent activation of D1 receptors was necessary for the induction of LTP in cortical pathways onto BLA principal neurons. Similarly, prior application of SCH23390 also blocked 5xHFS-induced LTP in BLA principle neurons (116±4% of baseline, n = 6, 5 animals, **Figure 2D**), Application of SCH23390 alone caused no enduring change in input resistance at any time point measured (F (10, 88) = 0.68, P = 0.7366, Two-way ANOVA). These results suggested that the LTP induced by TBS or 5xHFS share common receptor-effector signaling cascades.

To assess whether D1 receptor -mediated LTP in the BLA was dependent on activation of the adenylate cyclase (AC) – cAMP - protein kinase A (PKA) signaling cascade, we next tested the effect of inclusion in the patch pipette of a membrane-impermeable AC inhibitor, 2′, 5′-dideoxy-3′-ATP (0.5 µM), on 5xHFS-induced LTP. Intracellular application of 2′, 5′-dideoxy-3′-ATP significantly attenuated 5xHFS-induced LTP in BLA neurons (Control: 153±11% of baseline, n = 5; 2′, 5′-dideoxy-3′-ATP: 110±10% of baseline, n = 6; P<0.001). Similarly, intracellular application of the PKA inhibitor, KT5720 (100 nM), blocked not only the TBS-induced LTP (112±10% of baseline, n = 5, 4 animals, **Figure 2E**), but also the 5xHFS-induced LTP (107±7% of baseline, n = 5, 4 animals, **Figure 2F**). However, application of KT5720 caused no enduring change in input resistance at any time point measured (F (15, 98) = 0.26, P = 0.9761, Two-way ANOVA). Hence, the D1 receptor-dependent induction of LTP by either TBS or 5xHFS in BLA principal neurons was also dependent on activation of the AC – cAMP - PKA signaling cascade.

**Figure 3:**
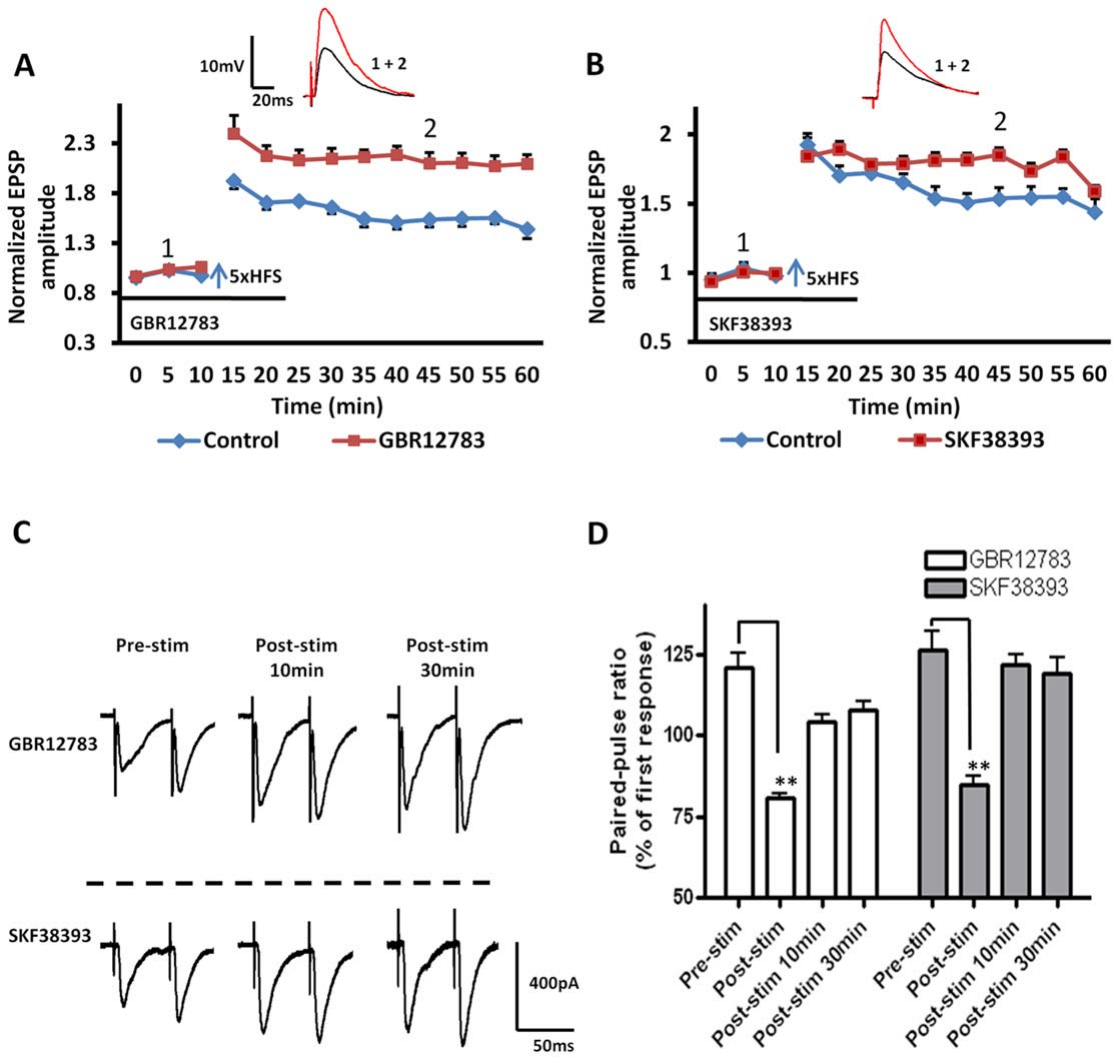
Application of either the dopamine transport inhibitor, GBR12783, or the D1 receptor agonist, SKF38393 facilitated 5xHFS induced LTP in BLA principal neurons. **A, B**) Bath application of either the selective dopamine uptake inhibitor, GBR12783 (10 µM, n = 6, blue diamonds), or the selective D1 family receptor agonist, SKF38393 (50 µM, n = 5, blue diamonds), before and during 5xHFS enhanced the magnitude of LTP in principal neurons. The time of drug application is marked by the *horizontal bar* below the line plots. **C**) Raw data showing the effects of drug application on the PPR of evoked EPSCs in BLA principal neurons. **D**) Bar chart showing the group data for the effects of drug application on the PPR. ** P<0.01. Error bars indicate S.E.M.

Having determined that LTP induction in BLA principal neurons was dependent on local dopamine release and subsequent activation of the D1 receptor - cAMP- PKA signaling cascade, we next examined whether the induction of LTP could be modified following bath application of the dopamine-specific monoamine transport inhibitor, GBR12783 (10 µM). Blockade of dopamine re-uptake with GBR12783 for 10 min prior to 5xHFS significantly enhanced the magnitude of LTP in all neurons tested compared to that induced in control ACSF (210±10% of baseline, n = 6, 4 animals, P<0.01, **Figure 3A**, red squares). Application of GBR12783 caused no enduring change in input resistance at any time point (F (10, 88) = 0.68, P = 0.7366, Two-way ANOVA). However, because application of GBR12783 enhanced both PTP (213±10% of baseline, 10 min following 5xHFS) and LTP, we next compared the PPR before, and at 10 and 30 min post of 5xHFS (**Figure 3C, D**). As illustrated in **Figure 3D** the PPR was not significantly different from control values 10 or 30 min after LTP induction (P <0.05), suggesting that the facilitation of PTP and LTP induced by GBR12783 was due to a facilitation of the postsynaptic response and not due to recruitment of an additional presynaptic component.

**Figure 4:**
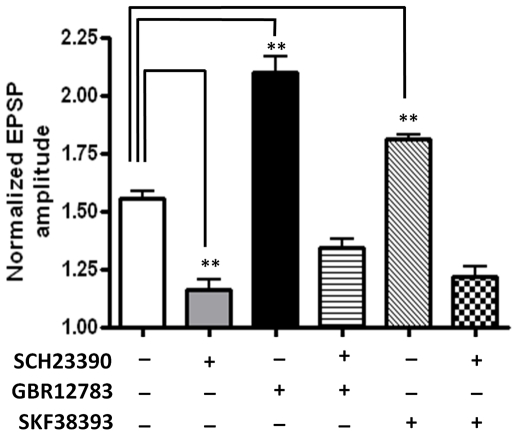
The D1 receptor antagonist SCH23390 fully blocked the induction of LTP by 5xHFS, as well as the facilitation of LTP by GBR12783 and SKF38393. The bar chart summarizes the group effects of different dopaminergic manipluations on the magnitude of LTP induced by 5xHFS. Prior application of SCH23390 (10 µM) blocked 5xHFS induced LTP (grey bar) as well as GBR12783 (n = 5, hooped bar) or SKF38393 (n = 5, stippled bar) facilitated LTP, ** P<0.01, * P<0.05. Error bars indicate S.E.M.

To ensure that blockade of dopamine re-uptake was not recruiting additional receptor subtypes; we then tested whether the enhancement of LTP was sensitive to D1 receptor antagonism. Pretreating slices for 10 min with SCH23390 (10 µM) fully blocked the GBR12783-enhanced LTP in all BLA principal neurons tested (134±4%, n = 5, 3 animals, P<0.001, **Figure 4**, hooped bar). Next, we tested whether exogenous application of the D1 receptor agonist, SKF38393 (50 µM), could also facilitate LTP. Pretreatment with SKF38393 for 10 minutes prior to 5xHFS significantly enhanced the magnitude of LTP compared to control (175±4% of baseline, n = 5, 3 animals, P<0.01, **Figure 3B**, red squares; **Figure 4**, diagonal bar). Application of SKF38393 alone caused no enduring changes in membrane input resistance (F (10, 110) = 0.58, P = 0.8302, Two-way ANOVA). Similarly, no change was observed in the PPR at 10 or 30 min after LTP induction in the presence of either GBR12783 or SKF38393 (**Figure 3D**). As expected, prior application of SCH23390 fully blocked the SKF38393-induced facilitation of LTP, and markedly attenuated the induction of LTP compared to control (122±4%, n = 5, 3 animals, P<0.001 **Figure 4**, hatched bar). Together these data suggested that release of endogenous dopamine and subsequent activation of D1 receptors was necessary for 5xHFS induction of LTP in BLA principal neurons, and that the magnitude of LTP was tightly regulated by the activity of the local dopamine-reuptake system.

**Figure 5:**
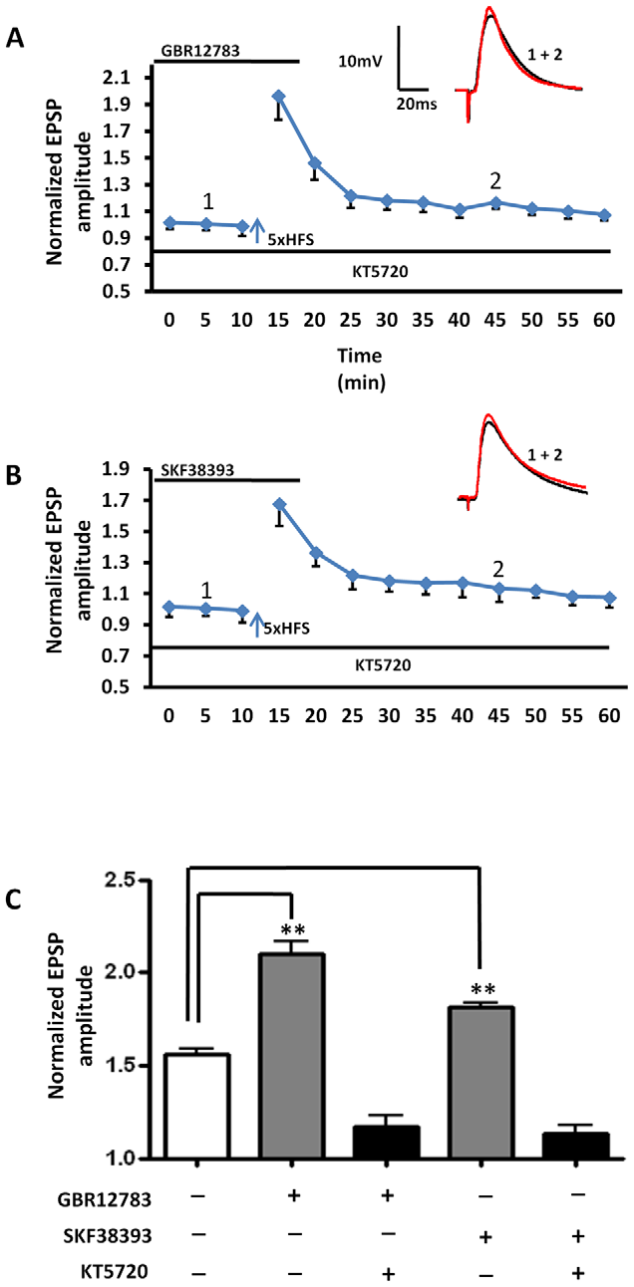
Facilitation of LTP by GBR12783 and SKF38393 are dependent on activation of the cAMP - PKA signaling cascade. **A**) Intracellular application of the PKA inhibitor, KT5720 (100 nM), blocked the GBR12783 facilitated LTP (n = 6) **B**) Application of KT5720 also fully blocked 5xHFS induced LTP in the presence of SKF38393 (n = 5) **C**) Bar chart showing the group data for drug effects 30 minutes post stimulation. *** P<0.001, Error bars indicate S.E.M.

Finally, addition of the AC inhibitor to the patch recording solution significantly attenuated the ability of both SKF38393 (SKF38393:179±9% of baseline, n = 5; SKF38393 + 2′, 5′-dideoxy-3′-ATP: 136±7% of baseline, n = 5; P<0.001) and GBR12783 (GBR12783: 208±13% of baseline, n = 5; GBR12783 + 2′, 5′-dideoxy-3′-ATP: 149±10% of baseline, n = 5; P<0.001) to facilitate LTP. Similarly, intracellular application of the PKA inhibitor, KT5720 (100 nM), blocked not only the GBR12783 facilitated LTP (116±5% of baseline, n = 6, 4 animals, **Figure 5A, C**), but also the SKF38393-induced facilitation of LTP (113±9% of baseline, n = 5, 3 animals, **Figure 5B, C**). Hence, D1 receptor activation appeared to facilitate LTP by acting through activation of the classic AC – cAMP - PKA signaling cascade and that this response was itself scaled by the level of extracellular dopamine.

### The role of BDNF in 5xHFS induction of LTP in BLA principal neurons

**Figure 6:**
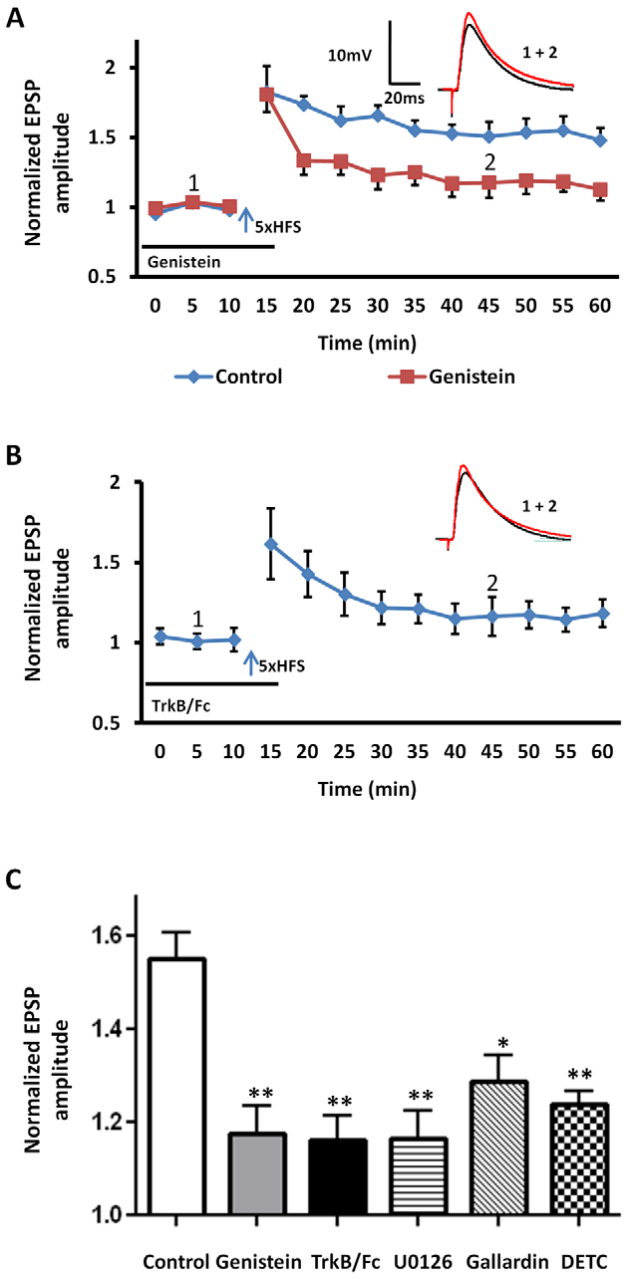
Activation of the BDNF and TrkB pathway is critical for the induction of LTP in BLA principal neurons. **A**) Bath application of of the non-selective receptor tyrosine kinase inhibitor, genistein (100 µM), before 5xHFS (arrow) blocked the induction of LTP in BLA principal neurons (n = 5, red squares). **B**) Bath application of the BDNF scavenger molecule, TrkB/Fc (1 µg/ml, n = 6), for 30 min before 5xHFS prevented the induction of LTP in BLA. **C**) Bar chart summarizing the group effects of modulating the BDNF and TrkB cascade on the induction of LTP. Intracellular application of MEK inhibitor, U0126 (10 µM, n = 5, hooped bar), for 30 min before recording abolished the induction of LTP. Similarly, bath application of the MMP inhibitor, GM6001 (Gallardin, 10 µM, n = 4, diagonal hashed bar), or the copper-zinc chelator (DETC, 20 µM, n = 5, stippled bar) significantly attenuated LTP in BLA principal neurons. ** P<0.01, * P<0.05. Error bars indicate SEM.

Like the dopaminergic system, a growing body of evidence suggests that fear learning is also dependent on local release of BDNF and subsequent activation of its cognate receptor, the TrkB receptor [14]. In the hippocampus, BDNF is released in response to HFS [29], and exogenous BDNF has been shown to facilitate field potential LTP in the BLA [15]. Hence, we next determined whether local release of endogenous BDNF may also contribute to 5xHFS-induced LTP in BLA principal neurons. As TrkB receptors are members of a family of receptor tyrosine kinases [30], we first examined the effects of bath application of the tyrosine kinase inhibitor, genistein (100 µM) on 5xHFS-induced LTP in BLA principal neurons. As shown in **Figure 6A**, bath application of genistein for 30 min prior to 5xHFS prevented the induction of LTP (118±6% of baseline, n = 5, 4 animals, P<0.01; **Figure 6A**, red squares, **Figure 6C**, grey bar), which is consistent with previous studies in the hippocampus [31]. Application of genistein caused no enduring change in input resistance at any time point measured (F (14, 150) = 0.31, P = 0.9921, Two-way ANOVA). However, the genistein effect could result from an action that was independent of BDNF - TrkB receptor activation. Hence, we next examined the effects on LTP induction of prior application of the TrkB-immunoglobulin-G fusion protein, TrkB-Fc (1 µg/ml), which scavenges BDNF and prevents TrkB receptor activation [32]. As shown in **Figure 6B**, prior application of TrkB-Fc prevented 5xHFS-induced LTP in BLA principal neurons (117±8% of baseline, n = 6, 4 animals, P<0.01; **Figure 6C**, black bar). Application of TrkB-Fc caused no enduring change in input resistance at any time point (F (12, 104) = 1.17, P = 0.3169, Two-way ANOVA).

Together these data suggest that 5xHFS of cortical inputs resulted, not only in the release of endogenous dopamine, but also endogenous BDNF release. TrkB receptors are coupled to three main signaling pathways, including the mitogen-activated protein (MAP) kinase kinase (MEK) cascade, phospho-inositide-3 kinase, and phospholipase C (PLC). In aplysia, activation of the MEK cascade is necessary for the induction of LTP and memory formation [33]. To explore whether activation of the MEK cascade downstream from BDNF was required for the induction of LTP in the BLA, we included the MEK inhibitor, U0126 (10 µM), in the patch recording pipette and allowed it to equilibrate for 30 min prior to LTP induction. Intracellular perfusion of U0126 prevented the induction of LTP in all neurons tested (116±5% of base line, n = 5, 4 animals; P<0.01; **Figure 6C**, hooped bar). Application of U0126 caused no enduring change in input resistance at any time point (F (10, 110) = 0.08, P = 0.9999, Two-way ANOVA).These results further suggested that BDNF release and TrkB receptor activation were also critically involved in the induction of LTP in BLA principal neurons, possibly acting via activation of the MEK cascade.

However, BDNF is synthesized as a precursor protein, preproBDNF, and proteolytic cleavage is required to generate mature BDNF (mBDNF) [34]. ProBDNF is processed into mBDNF by either matrix metalloproteinases (MMPs) [35] or by tissue plasminogen activator (tPA)-dependent activation of plasminogen [36]. A recent study reported that tPA was almost completely absent in the BLA [37]. Hence, we reasoned that if LTP induction in the BLA was dependent on TrkB receptor activation, then the conversion of proBDNF to mBDNF by MMPs could play a significant role in this process. Thus, blockade of MPPs would be predicted to attenuate LTP. Consistent with this prediction, application of the non-selective MMP inhibitor, gallardin (10 µM), 30 min before 5xHFS significantly reduced the magnitude of LTP to 131±7% of baseline (n = 4, 3 animals, P<0.05; **Figure 6C**, diagonal bar), which is consistent with previous studies in MMP-2 knockout mice [38]. Application of gallardin caused no enduring change in input resistance at any time point (F (12, 104) = 0.6, P = 0.8370, Two-way ANOVA). Significantly, MMPs are only activated in the presence of extracelluar zinc [39], and cortical afferents to the BLA are known to contain vesicular zinc [40]. Hence, zinc released from presynaptic terminals could directly activate MMPs in the BLA following 5xHFS and trigger the conversion of proBDNF to mBDNF. Consistent with this observation, inclusion of the copper-zinc chelator (DETC, 20 µM) in the ACSF significantly attenuated 5xHFS-induced LTP in BLA principal neurons (123±6% of base line, n = 5, 4 animals, P<0.01, **Figure 6C**, hatched bar). Whereas application of DETC alone caused no enduring change in input resistance at any time point measured (F (13, 112) = 0.70, P = 0.6963, Two-way ANOVA). Moreover, as shown below, DETC failed to block the facilitating effects of exogenous BNDF application on 2xHFS induced LTP, suggesting that DETC application was not affecting LTP-induction independently of TrkB receptor activation. Together these data suggested that 5xHFS of cortical inputs into the BLA may cause a concomitant release of dopamine, zinc, and BDNF resulting in the subsequent activation of postsynaptic D1 and TrkB receptors, both of which act to enhance the induction and maintenance of LTP.

### Gain-control of LTP induction in BLA principal neurons

**Figure 7:**
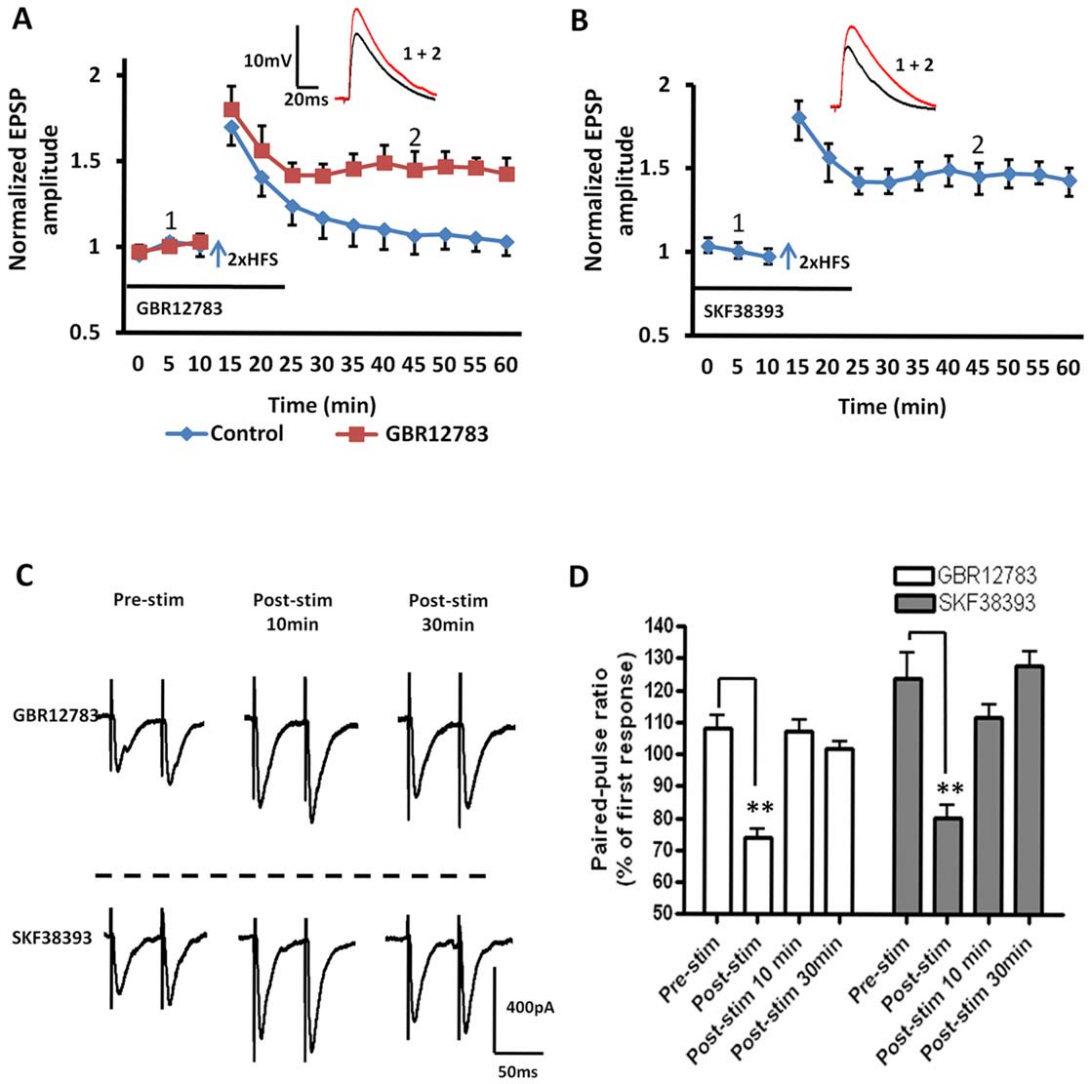
D1 receptor activation acts to reduce the threshold for LTP induction in BLA principal neurons. **A**) Application of the dopamine reuptake inhibitor GBR12783 (10 µM, n = 5), or **B**) the D1 receptor agonist SKF38393 (50 µM, n = 5) induced a robust and stable LTP in BLA principal neurons following 2xHFS. **C**) Raw data showing the effects of GBR12783 and SKF38393 on the PPR in BLA principal neurons. **D**) A bar chart illustrating the group effects of drug application on the PPR of EPSCs before, immediately after, and at 10 min and 30 min post 2xHFS. ** P<0.01. Error bars indicate S.E.M.

**Figure 8:**
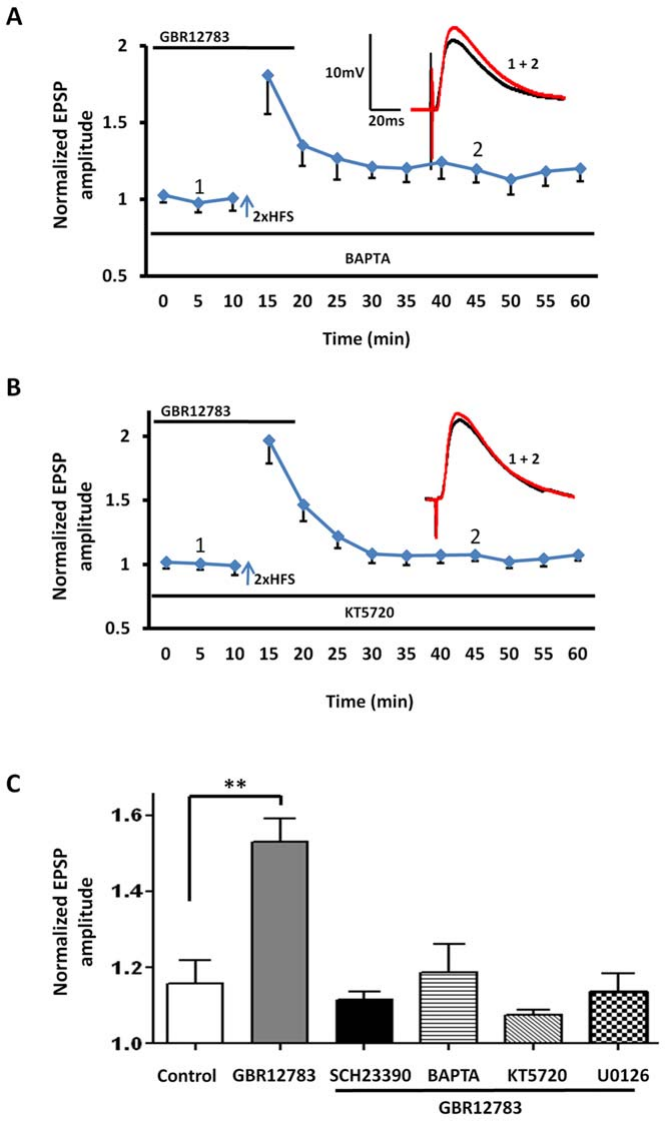
D1 receptor-dependent reduction of the threshold of LTP induction requires calcium influx, and induction of the cAMP-PKA and MEK-MAPK signaling cascades. **A**) Inclusion of the calcium chelator, BAPTA (10 mM) in the patch pipette (n = 5) abolished the GBR12783 mediated LTP induction by 2xHFS. **B**) Inclusion of the PKA inhibitor, KT5720 (100 nM) (n = 5) also blocked the GBR12783 mediated LTP induction by 2xHFS. **C**) Bar chart summarizing the group effects of GBR12783 on LTP induction using 2xHFS. As illustrated, pretreatment with SCH23390 (black bar), BAPTA (hooped bar), KT5720 (diagonal bar), and U0126 (hatched bar) all fully blocked the GBR12783-facilitated LTP (n = 5 in each case). ** P<0.01. Error bars indicate SEM.

Elsewhere in the brain, D1 receptor activation or BDNF release has been shown to lower the threshold for LTP induction [41], [42]. Hence, we next determined whether activation of D1- or TrkB receptors could affect the threshold for LTP induction in the BLA using the 2xHFS stimulation protocol, which could not induce LTP in principal neurons (see **Figure 1B**). Significantly, in the presence of either the dopamine transport inhibitor, GBR12783 or the D1 receptor agonist, SKF38393, 2xHFS induced a robust and stable LTP in BLA principal neurons (GBR12783: 153±8% of baseline, n = 5, 4 animals, P<0.01; **Figure 7A**, red squares, and **Figure 8C**, grey bar; SKF38393:148±6% of baseline, n = 5, 4 animals, P<0.01; **Figure 7B**). Moreover, in all BLA principal neurons tested pretreating slices for 10 min with the D1 receptor antagonist SCH23390 (10 µM) fully blocked the GBR12783 (111±4% of baseline, n = 5, 3 animals, P<0.001, **Figure 8C**, black bar) and SKF38393-mediated facilitation of LTP induced by 2xHFS (107±5% of baseline, n = 5, 3 animals, P<0.001, data not shown). However, as illustrated in **Figure 7C**, analysis of the PPR showed no significant difference from control values at 10 or 30min after 2xHFS LTP induction in the presence of either GBR12783 or SKF38393 (**Figure 7C, D**) suggesting that facilitation of LTP was not dependent on alterations in presynaptic release mechanisms.

Consistent with this observation, inclusion of either BAPTA (10 mM) (119±8% of baseline, n = 5, 3 animals, P<0.05, **Figure 8A, C**, hooped bar) or the PKA inhibitor KT5720 (100 nM) (106±10% of baseline, n = 5, 3 animals, P<0.001, **Figure 8B**, C, diagonal bar) in the patch pipette fully blocked the induction of LTP by 2xHFS in the presence of GBR12783. Together these data suggested that the postsynaptic mechanisms underlying the induction of LTP by 2xHFS in the presence of GBR12783 were similar to those mediating the response to 5xHTS. Significantly, application of the MEK inhibitor, U0126, also blocked LTP induced by 2xHFS in the presence of GBR12783 (114±5% of baseline, n = 5, 3 animals, P<0.05, **Figure 8C**, hatched bar), suggesting that activation of the BDNF-TrkB pathway may also play a role in the induction of LTP by 2xHFS in the presence of GBR12783.

**Figure 9:**
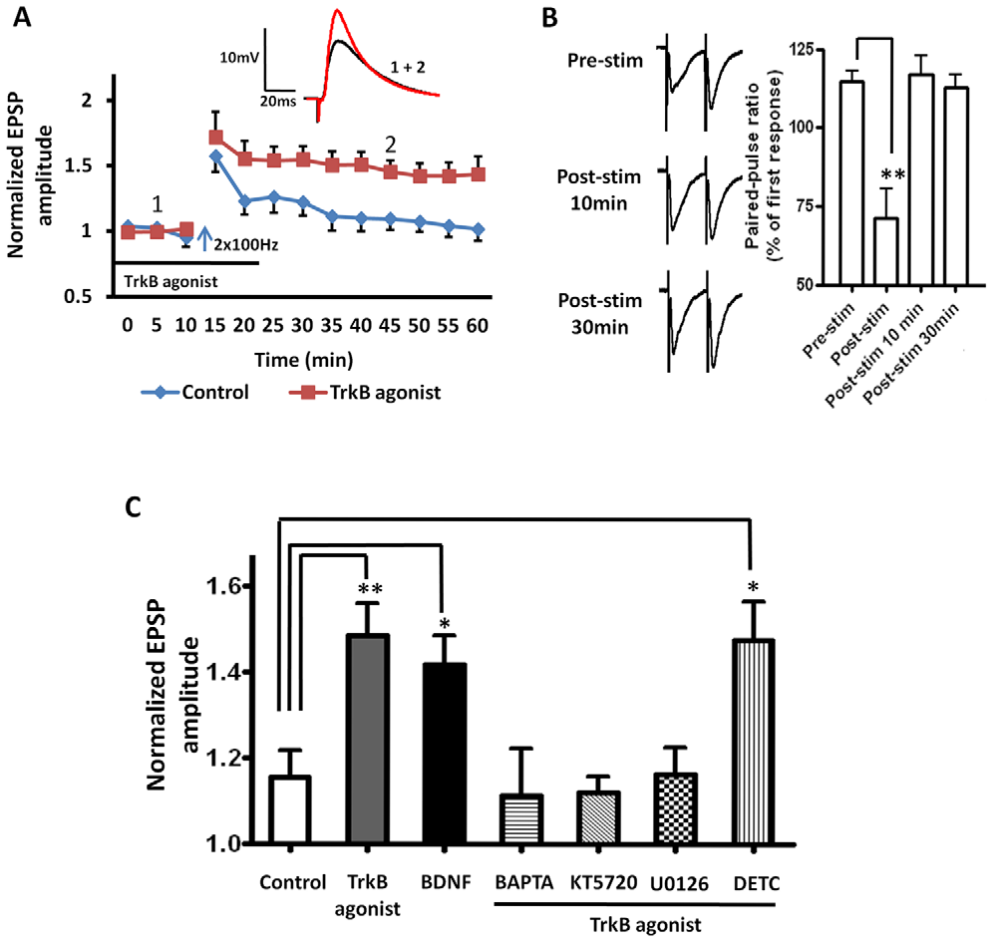
TrkB receptor activation lowers the threshold of LTP induction in the BLA. **A**) Application of the TrkB receptor agonist, 7, 8-dihydroxyflavone (500 nM, n = 6) facilitated the induction of LTP by 2xHFS. **B**) Left, raw data showing a typical PPR response before, and at 10 and 30 min after 2xHFS in the presence of the TrkB agonist. Right, a bar chart illustrating the group data. **C**) Bar chart summarizing the group effects of TrkB receptor activation on LTP threshold using 2xHFS. Application of BDNF (100 ng/ml, n = 4) for 30 min, mimicked the induction of LTP by 2xHFS induced by 7, 8-dihydroxyflavone. Intracellular application of calcium chelator, BAPTA (10 mM, n = 5, hooped bar) as well as the PKA inhibitor, KT5720 (100 nM, n = 5, diagonal bar) or the selective MEK inhibitor, U0126 (10 µM, n = 4, hatched bar) fully blocked the 7, 8-dihydroxyflavone facilitated LTP. However, incubation with DETC (20 µM, n = 6, striped Bar) did not block this effect. ** P<0.01, * P<0.05. Error bars indicate SEM.

Consistent with this hypothesis, prior activation of TrkB receptors with either the TrkB receptor agonist, (7, 8-dihydroxyflavone, 500 nM), or BDNF (100 ng/ml), reduced the threshold for LTP-induction in response to 2xHFS (7, 8-dihydroxyflavone: 149±8% of baseline, n = 6, 5 animals, P<0.01, **Figure 9A**, red squares, and **Figure 9C**, grey bar; BDNF: 142 ±9% of baseline, n = 4, 4 animals, P<0.01, **Figure 9C**, black bar). Application of either the TrkB receptor agonist or BDNF alone caused no enduring changes in input resistance at any time point measured (7, 8-dihydroxyflavone: (F (10, 110) = 1.44, P = 0.1739; BDNF: (F (13, 112) = 0.17, P = 0.9995 Two-way ANOVA)). Similar to the GBR12783 response, intracellular application of BAPTA (10 mM) (111±11% of baseline, n = 5, 3 animals, P<0.05, **Figure 9C**, hooped bar) or KT5720 (100 nM) (112±4% of baseline, n = 5, 4 animals, P<0.01, **Figure 9C**, diagonal bar) fully blocked the 2xHFS induced LTP in the presence of the TrkB agonist. Hence, facilitation of LTP induction in BLA principal neurons by D1 and TrkB receptor activation appear to share a common pathway that is dependent on postsynaptic calcium influx and activation of the cAMP -PKA signaling cascade. Significantly, intracellular perfusion of the MEK inhibitor U0126 (10 µM) also prevented the facilitation of LTP by the TrkB receptor agonist, 7, 8-dihydroxyflavone, (116 ±6% of baseline, n = 4, 3 animals, P<0.05, **Figure 9C** hatched bar), suggesting that activation of D1 and TrkB receptors may converge at the level of MEK to facilitate LTP. In contrast, application of the zinc chelator, DETC, failed to block the TrkB agonist effect (148 ±9% of baseline, n = 6, 5 animalsP<0.01, **Figure 9C** striped bar), suggesting that exogenous TrkB receptor activation can lower the threshold of LTP induction in the BLA through direct activation of the MEK cascade, and that this effect is independent of stimulus-induced zinc release.

**Figure 10:**
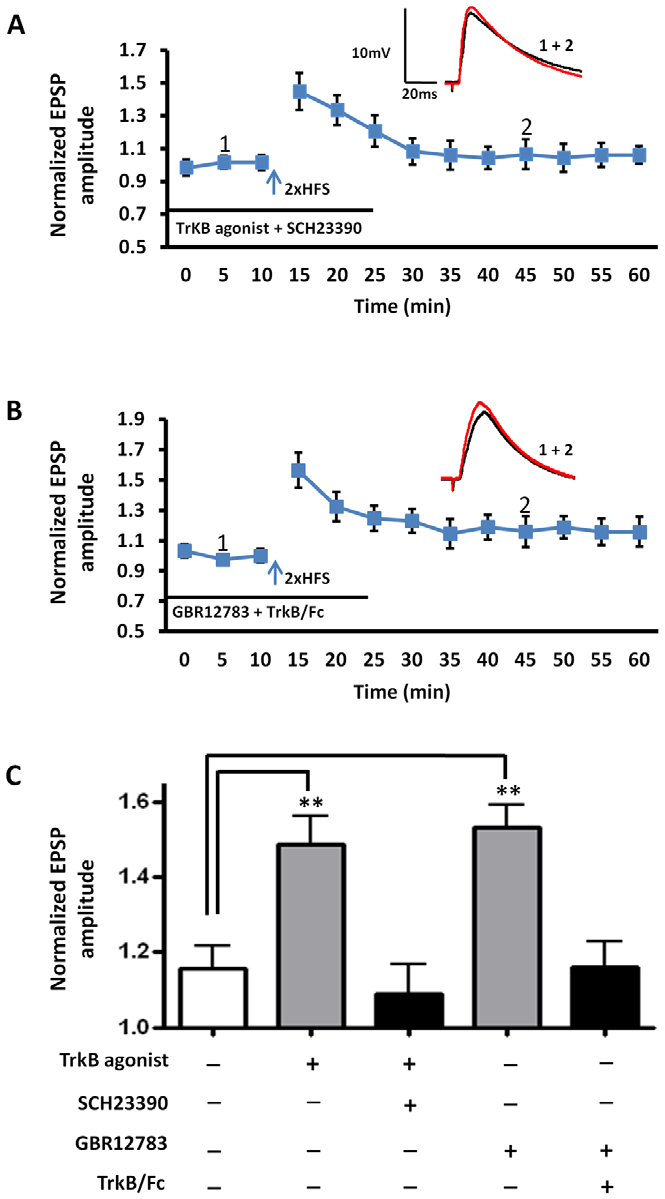
Synergistic activation of D1- and TrkB receptors lowers the threshold for LTP induction in BLA principal neurons. **A**) Preincubation with SCH23390 (10 µM) blocked the induction of LTP by 2xHFS in the presence of 7, 8-dihydroxyflavone (500 nM). **B**) Preincubation with TrkB/Fc blocked the induction of LTP by 2xHFS in the presence of GBR12783. **C**) Bar chart illustrating the synergistic interaction between D1 and TrkB receptors to reduce LTP threshold in BLA principal neurons. ** P<0.01. Error bars indicate S.E.M.

Because both D1- and TrkB receptor activation lowered the threshold for LTP induction in the BLA, and D1 receptor antagonism completely abolished LTP in response to 5xHFS, we next investigated whether these two transmitter systems may act synergistically to facilitate LTP induction. Here, the D1 receptor antagonist, SCH23390, was applied 10 min before 7, 8-dihydroxyflavone, which was applied 20 min prior to 2xHFS. Prior application of SCH23390 fully blocked the ability of the TrkB receptor agonist to lower the LTP threshold in response to 2xHFS (109 ±8% of baseline, n = 6, 5 animals, **Figure 10A, C**), suggesting that a synergistic activation of D1 and TrkB receptors was necessary to reduce LTP threshold. To examine if the reverse relationship applied, we next examined the effects of prior application of the BDNF scavenger, TrkB-Fc, on the facilitation of LTP induced by 2xHFS in the presence of the dopamine transport inhibitor, GBR12783. A 10 min pretreatment with TrkB-Fc fully blocked the GBR12783-induced lowering of LTP threshold (114±9% of baseline, n = 5, 4 animals, **Figure 10B, C**), further supporting the hypothesis that a synergistic interaction between these two transmitter systems was required to reduce LTP threshold in BLA principal neurons.

**Figure 11:**
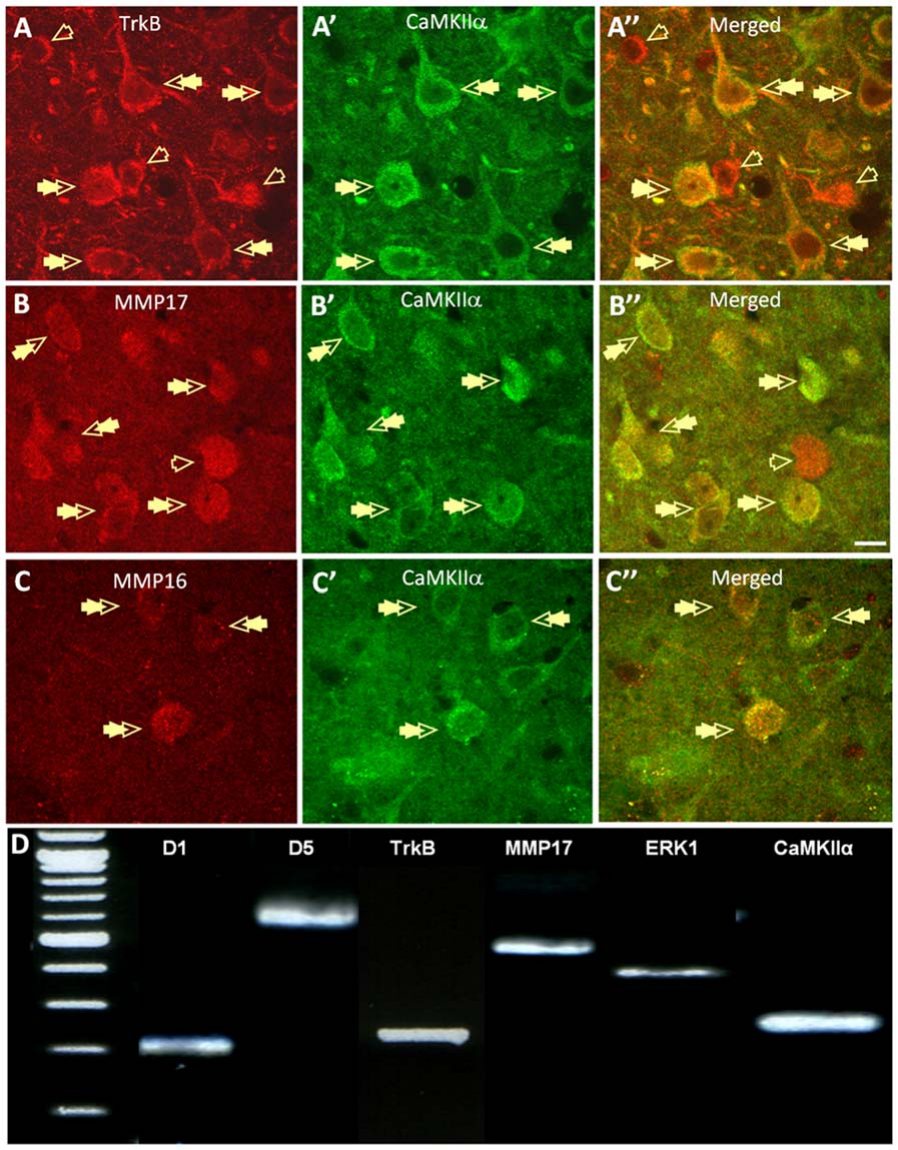
BLA projection neurons co-express mRNA and protein for D1- and TrkB receptors, as well as MMP17. A-A″) Photomicrographs showing the high level of co-localization for TrkB (A, red) and CaMKIIa- (A′, green) immunoreactivity in putative principal neurons of the BLA (A″, merged, double arrows). Note, a subpopulation of TrkB-positive neurons do not co-localize CaMKIIa (A″, open arrows, merged). B-B″) Photomicrographs showing the high level of co-localization for MMP17 (B, red) and CaMKIIa- (B′, green) immunoreactivity in BLA neurons (B″, merged, double arrows). Note, a subpopulation of MMP17-positive neurons do not co-localize CAMKIIa. C-C″) Photomicrographs showing the co-localization of MMP16 (C, red) and CaMKIIa (C′, green) immunoreactivity in BLA neurons (C″, merged, double arrows). Note, a subpopulation of CAMKIIa-positive neurons do not co-localize MMP16. All photomicrographs were taken from the basolateral nucleus of the BLA. In all images magnification  = 63x, scale bar  = 10 mm. C) Photograph of an agarose gel showing the typical mRNA expression pattern for a D1- and D5- mRNA-positive principal neuron. The associated table shows the relative expression of D1, D5, TrkB, MMP17 and ERK_1/2_ mRNA expression in single BLA principle neurons.

Finally, we ran immunohistochemical- and single-cell RT-PCR studies to confirm that all of the proteins predicted by our physiological studies were expressed in the BLA. We have shown previously that D1 receptors are found in the spines of BLA principal neurons [7], where they form a close association with NMDA receptors [8], and the Allen Brain Atlas shows moderate to high mRNA expression levels for the matrix metalloproteinase isoforms, MMP16 and MMP17, and also the TrkB receptor. Here, we extend these observations using dual-immnuofluorescence microscopy to show that protein for MMP16, MMP17, and the TrkB receptor are extensively co-localized with CaMKIIα, a major component of excitatory synapses in presumed BLA principal neurons (**Figure 11A–C**).

To further confirm that neurons of the BLA express the machinery necessary to mediate the synergistic modulation of LTP induction by D1- and TrKB receptors, we extracted cytosolic mRNA from physiologically identified BLA principal neurons and performed single-cell RT–PCR to examine their mRNA expression profile. As expected, all cells sampled (n = 15) expressed mRNA transcripts for CaMKIIα. However, 7/15 neurons sampled expressed only D_1_ receptor mRNA transcripts, 5/15 neurons expressed both D_1_ and D_5_ receptor mRNA transcripts, and 3/15 neurons expressed only D_5_ receptor mRNA transcripts. Intriguingly, all of the neurons that expressed D_1_ receptor mRNA transcripts also expressed mRNA transcripts for the TrkB receptor as well as ERK_1/2_. Moreover 8/15 neurons expressed MMP17 transcripts and 3/15 neurons expressed MMP16 mRNA transcripts. Significantly, those neurons that expressed both D_1_ and D_5_ receptor mRNA transcripts expressed all of the proteins necessary to facilitate a synergistic interaction between the D1 and BDNF signaling cascades (**Figure 11D**).

## Discussion

To the best of our knowledge, this study represents the first of its kind to show that a) endogenous dopamine release and subsequent D1 receptor activation is prerequisite for long-term potentiation of glutamatergic input onto BLA principal neurons from cortical afferents, b) that endogenous release of dopamine, zinc, and BDNF results in a synergistic activation of D1 and TrkB receptors, which together function to lower the threshold for LTP induction, and that c) convergent activation of the MEK-ERK signaling cascade may play a critical role in lowering the threshold for LTP induction.

We believe that the results of our study offer a plausible mechanism by which the results of several previously independent studies can be unified into a common model of affective memory formation in the BLA. For example, previous studies have shown that the majority of dopaminergic terminals in the BLA make synaptic contacts onto the spines of principal neurons [8], that these spines co-localize D_1_- and NMDA receptors, which function to modulate excitatory synaptic transmission [7], and that local dopamine release and subsequent activation of D1 receptors is necessary for the acquisition of fear memory [3]. Similarly, Bolshakov, Shumyatsky and co-workers have shown that synaptically released zinc not only regulates LTP formation in the BLA but also fear memory formation [43], [44]. Likewise, BDNF release and the subsequent activation of TrkB receptors in the BLA has been shown to play a critical role in regulating synaptic plasticity and fear memory formation [43], [45].

In the present study, we have shown that LTP can be induced by either TBS or by spaced 5xHFS. Studies in the hippocampus have shown that spaced HFS and TBS-induced LTP are cAMP-PKA dependent [23], [24], [25]. Consistent with these data, we have shown that in BLA principal neurons TBS- and 5xHFS-induced LTP were both blocked by the D1 receptor antagonist SCH23390, as well as by intracellular application of adenylate cyclase and PKA inhibitors. Moreover, our paired pulse experiments showed that the PPR was not altered after LTP induction, suggesting that both TBS- and spaced 5xHFS-induced LTP may be dependent on activation of the same postsynaptic cAMP-PKA signaling cascade but independent of presynaptic modifications. In the hippocampus, Kim and colleagues have shown that LTP induced by 4xHFS at 40s intervals was not blocked by a PKA inhibitor [46]. The reason(s) for the difference between their results and ours are not immediately apparent but could be attributable to differences in the recording method; whole-cell patch clamp recording compared to field potential recording, or may simply reflect differences in the sensitivity to LTP induction between the BLA and hippocampus.

We have shown that D_1_ receptors co-distribute with the NMDA receptor NR1 subunit in the spines of BLA principal neurons, where they function to modulate NMDA currents [8]. The co-activation of these two receptors has been shown to up-regulate the surface expression and incorporation of the GluR1 receptor subunit of the AMPA receptor at synaptic sites in hippocampus [47]. Thus, concurrent activation of D_1_ and NMDA receptors alone could functionally modulate the D_1_ receptor induction of LTP in BLA principal neurons. Moreover, in this study we have shown that inhibition of AC and PKA activity can block TBS- or 5xHFS-induced LTP as well as the facilitation of LTP induced by the dopamine transport inhibitor, GBR12783 using 2xHFS, suggesting that both the induction and facilitation of D1-induced LTP in BLA principal neurons is dependent on activation of the AC – cAMP -PKA signaling cascade. These results are consistent with the seminal studies of Schafe and LeDoux showing that fear memory consolidation in the amygdala is a cAMP- and PKA-dependent process [48], [49], Significantly, Jentsch and Taylor have shown that stimulation of PKA activity in the amygdala facilitates reward-related learning [50], further suggesting that aversive and appetitive learning may share common sub-cellular substrates in the BLA.

Although our results suggest that D1 receptor activation alone may be sufficient to enable the induction of LTP in principal neurons, synaptic release of zinc together with activation of D1 and TrkB receptors might not occur independently of each other. Hence, stimulation-induced BDNF activation of TrkB receptors appears to be critically dependent on synaptically released zinc, concurrent activation of D1- and TrkB receptors would then act synergistically to markedly facilitate the induction of LTP in the BLA. Indeed, in cortical networks cross-talk between the D1 system and the BDNF system may be the rule, rather than the exception. Hence, Cammarota and colleagues have reported that a similar interaction regulates long-term memory (LTM) formation in the hippocampus. Here, infusion of the D1 receptor antagonist, SCH23390, blocked training-induced increase in BDNF and prevented LTM. Conversely, infusion of the D1 receptor agonist, SKF38393, increased BDNF levels and facilitated LTM. Significantly, the D1 mediated effect was blocked by infusion of a PKA inhibitor, and infusion of a BDNF function-blocking antibody impaired the SKF38393-caused increase in LTM [19], suggesting that hippocampal-dependent LTM requires a synergistic interaction between these two systems and activation of the AC-cAMP-PKA cascage. Our results could provide insight into the cellular substrate mediating this cross-talk.

Moreover, we have shown that a key component potentially linking synaptically released zinc and concurrent activation of D1- and TrkB receptors is the functional expression of extracellular matrix metalloproteinase (MMPs) by principal neurons of the BLA. MMPs are not constitutively active under basal conditions, and require synaptically released zinc to bind as a co-factor before they become fully active [37], [39]. Synaptic concentrations of zinc can reach micromolar levels following HFS [50], suggesting that extracellular MMPs in the BLA may be preferentially activated only in response to strong (salient) sensory input. A similar activity-dependent conversion of pro-BDNF to mBDNF has been reported in cultured hippocampal neurons [51]. Here, low-frequency stimulation was reported to release proBDNF [51]. In contrast, following HFS substantial amounts of mBDNF were detected [29], suggesting that a co-factor may be released during HFS that facilitates the conversion of proBDNF to mBDNF.

Significantly, local release of zinc is restricted to a subset of glutamatergic afferents arising from cortical/limbic structures, and not from sub-cortical structures such as the thalamus, whose terminals are devoid of synaptic zinc [50] . Recently, Humeau and colleagues reported that simultaneous stimulation of cortical and thalamic afferents onto neurons of the lateral amygdala (LA) induced heterosynaptic LTP at cortical, but not at thalamic, inputs [27]. Consistent with this observation, we were unable to induce robust LTP in the thalamic pathway using stimulation parameters that consistently induced LTP in the cortical input onto BLA principal neurons. However, a presynaptic form of LTP in the thalamic input onto LA neurons has been reported using a prolonged period of paired-pulse stimulation [52]. Further studies are required to determine if a similar mechanism exists in the thalamic input onto principal neurons of the BLA. Nonetheless, our data suggest that concurrent activation of cortical and thalamic inputs into the BLA, as occurs during conditioned- (CS) and unconditioned-stimulus (US) pairing in fear conditioning, may selectively restrict BDNF-dependent LTP to the site of cortical, sensory, input. Thus, presynaptic zinc release from the cortical input may contribute to the maintenance of a precise sensory topography for LTP induction (synaptic tagging) even within the dendritic arbor of an individual principal neuron, thereby preventing sensory generalization.

Synaptically released zinc has been shown to affect multiple postsynaptic receptors and ion channels [50], and is able to transactivate TrkB receptors in the hippocampal mossy fiber-CA3 pyramid synapse [53]. Hence, it is possible that the inhibition of LTP observed following chelation of zinc by DETC may result from processes that were independent of the inhibition of MMP activation. However, the similarity of the response to DETC, gallardin, and TrkB/Fc application, together with the inability of DETC pretreatment to block the response to exogenous TrkB receptor agonist application strongly suggests that DETC was acting to directly block the activation of MMPs and, hence, prevent the conversion of proBDNF to mBDNF and subsequent activation of TrkB receptors. Consistent with this observation MMPs have been shown to play a key role in facilitating synaptic plasticity in the hippocampus and prefrontal cortex [54], [55]. Importantly, our immunohistochemical and single cell RT-PCR study revealed that principal neurons that express D_1_ and D_5_ receptor mRNA also show high protein expression for both membrane-type matrix metalloproteinase 16 and 17 (MMP16, MMP17), as well as the TrkB receptor. It is possible that either MMP16 or MMP17 and the TrkB receptor may be co-localized in the spines of principal neurons to facilitate BDNF-induced modulation of sensory input. LTP could be induced in almost all of the projection neurons that we recorded and, hence, MMP16 and MMP17 may serve the same function in different populations of principal neurons. Moreover, principal neurons of the BLA may be capable of differentially expressing multiple subtypes of MMPs, each of which could facilitate LTP induction. Finally, principal neurons of the BLA also have a random spatial orientation and an extensive, overlapping, dendritic arbor. Following HFS, zinc released from presynaptic cortical terminals may activate MMPs in those neurons that express them, which could, in turn, modulate the conversion of proBDNF to mBDNF even at synaptic contacts where the postsynaptic neuron does not express MMPs. Thus zinc release from limbic afferents may ensure input specificity for LTP induction but may also allow generalization of the response to clusters of neighboring principal neurons.

Our results showing a gain of function effect, *i.e.* reduced LTP threshold, in the synaptic response to sub-threshold stimulation following application of BDNF or the dopamine transport inhibitor, GBR12783, are also consistent with previous studies in the prefrontal cortex and hippocampus. Here, endogenous DA and BDNF have been shown to amplify LTP via activation of D1 receptors [10], and TrkB receptors [56] respectively. A similar dopamine D1 receptor-dependent gain of sensitivity has also been reported for spike-timing-dependent plasticity in CA1 hippocampal neurons [57]. However, our study extends these observations to demonstrate that, in the BLA, the gain of function effect in response to DA or BDNF is dependent on the concurrent activation of both receptor-effector cascades. Moreover, our results further suggest that concurrent activation of D1 and TrkB receptors may trigger second messenger cascades that converge at the level of the MEK-ERK pathway.

We have shown that the D1 receptor-mediated facilitation of LTP induction in BLA principal neurons is critically dependent on the activation of the AC – cAMP - PKA signaling cascade. Recently, BDNF-dependent learning in the BLA was shown to be mediated by activation of the MAPK and PI3K signaling pathways, and abolished in the presence of MEK inhibitors [15]. Consistent with this observation, we have shown that the MEK inhibitor, U0126, abolished both 5xHFS-induced LTP as well as the gain of function effect induced by either exogenous TrkB agonist or GBR12783 application. Significantly, Gean and colleagues have reported that PI3K acts as a critical intermediary for PKA-induced MAPK activation in the amygdala, and is required for amygdala LTP and fear memory formation [58]. TrkB receptor activation can activate PI3K and, hence, concurrent activation of D1 and TrkB receptors may lower the threshold of LTP induction by convergent activation of the MEK-MAPK signaling cascade. Although our study did not examine PLCγ activation, Musumeci and colleagues have recently shown that mutation of the PLCγ docking site of the TrkB receptor also impairs acquisition of fear conditioning, suggesting that synergism with the PLC cascade may also lower the threshold for LTP induction [17].

In summary, our results show that following HFS synaptically released zinc facilitates the synergistic interaction between endogenously released dopamine and BDNF to control the magnitude of LTP in BLA principal neurons. Aberrant activity of the dopaminergic- and BDNF systems have been implicated in the psychopathology of psychiatric disorders such as post-traumatic stress disorder (PTSD) and depression [59], as well as drug abuse [60]. Our observations on the synergistic interaction between dopamine and BDNF and their ability to amplify sensory input may provide important clues to the molecular mechanisms underlying the development of sensory generalization in PTSD, and sensitization in addiction.

## Methods

### Animals

Male Sprague–Dawley rats (5–7 weeks old, Charles River, Raleigh, NC, USA) were used in this study. Every effort was used to minimize both animal suffering and the number of animals necessary to complete the study. All experimental protocols strictly conform to National Institutes of Health guidelines for the Care and Use of Laboratory Animals, and were approved by the Institutional Animal Care and Use Committee of Emory University.

### Slice preparation

Slices of 350 µm thickness containing the BLA were obtained as described previously [61]. Briefly, the brains of isoflurane (Fisher Scientific, Hanoverpark, IL, USA) anesthetized animals were rapidly dissected and immersed in a cold (4°C) 95–5% oxygen/carbon dioxide oxygenated “cutting solution” with the following composition (in mM): NaCl (130), NaHCO3 (30), KCl (3.50), KH2PO4 (1.10), MgCl2 (6.0), CaCl2 (1.0), glucose (10), supplemented with kynurenic acid (2.0). Slices containing the BLA were cut using a Leica VTS-1000 Vibratome (Leica Microsystems Inc., Bannockburn, IL, USA). After cutting, slices were maintained at 37°C in oxygenated “cutting solution” for at least 50 min before transferring to regular artificial cerebrospinal fluid (ACSF) containing (in mM): NaCl (130), NaHCO3 (30), KCl (3.50), KH2PO4 (1.10), MgCl2 (1.30), CaCl2 (2.50), and glucose (10). Slices were kept in the regular ACSF for at least 30 min before recording.

### Patch clamp recording

Individual slices were then transferred to a submersion-type recording chamber mounted on the fixed stage of a Leica DMLFS microscope (Leica Microsystems Inc., Bannockburn, IL, USA), and continuously perfused by gravity-fed oxygenated 32°C ACSF at a flow rate of 1–2 ml/min. Slices were viewed using differential interference contrast (DIC) optics and infrared (IR) illumination with an IR sensitive CCD camera (Orca ER, Hamamatsu, Tokyo Japan). Thin-walled borosilicate glass patches electrodes (WPI, Sarasota, FL, USA) which had a resistance of 4–6 MΩ were filled with (in mM): 130K-gluconate, 2 KCl, 10 HEPES, 3 MgCl2, 2 K-ATP, 0.2 NaGTP, and 5 phosphocreatine, adjusted to pH 7.3 with KOH, and having an osmolarity of 280–290 mOsm. Individual BLA projection neurons were visualized in situ using DIC microscopy in combination with a 40x water immersion objective and displayed in real time on a computer monitor. Projection neurons were identified according to their characteristic size and shape [62], and were normally located between 50 and 120 µM beneath the surface of the slice. Data acquisition and analysis were performed using a MultiClamp700B amplifier in conjunction with pClamp10.0 software and a DigiData 1320A AD/DA interface (Molecular Devices, Burlingame, CA, USA). Whole cell patch clamp recordings were obtained and recorded voltages were low-pass filtered at 5 kHz and digitized at 10–20 kHz.

At the start of each experiment, a series of standardized current clamp protocols were performed to further validate the identity of BLA projection neurons [63]. Excitatory postsynaptic potentials (EPSPs) onto BLA projection neurons were evoked as previously described [64]. In brief, a concentric bipolar stimulation electrode (FHC, Bowdoinham, ME, USA) was placed approximately 500 µm from the recorded neuron, close to the fiber tract of the external capsule immediately adjacent to the BLA. In all experiments, 50 µM picrotoxin was added to the patch solution to block GABA_A_ currents exclusively in the recorded neuron. Slices were continuously perfused with oxygenated ACSF (32°C) containing the GABA_B_ receptor antagonist CGP36742 (5 µM). This recording configuration allowed stable recording of isolated EPSPs without contamination from epileptiform, recurrent EPSPs.

EPSPs (adjusted to 30% of maximal response) were evoked at 0.05 Hz, a 10 min baseline period was recorded in each experiment, and recordings continued for at least 40min after LTP induction. Initial EPSP amplitudes were normalized to the average of the baseline EPSP amplitude. 5x high frequency stimulation (HFS) LTP , an NMDA-receptor-dependent form of LTP [65] consisted of five trains of stimulation at 100 Hz for 1-s duration, applied with 20 s intervals; 2xHFS consisted of only two, 1-s trains of 100 Hz stimulation. Theta burst stimulation (TBS) consisted of 40 ms duration, 100 Hz bursts delivered at 5 Hz for 5 s (25 bursts with 4 pulses per burst, for a total of 100 pulses. A DC holding current was injected to maintain the membrane potential at -70 mV, except during HFS or TBS when the potential was adjusted to -60 mV to facilitate spike firing.

To calculate the paired-pulse ratio (PPR) two EPSCs were evoked with an inter-stimulus-interval of 50 ms, PPR was calculated as (eEPSC_1_/eEPSC_2_), where eEPSC_1_ and eEPSC_2_ represent the amplitude of the first and the second eEPSC, respectively. PPR was measured before, immediately after, as well as 10 min and 30 min after each of the LTP induction protocols (TBS, 5xHFS and 2xHFS).

### Drug application

The following drugs were obtained from (1) Sigma-Aldrich (St. Louis, MO, USA): picrotoxin, 3-[[(3, 4-Dichlorophenyl) methyl] amino] propyl]diethoxymethyl) phosphinic acid (CGP52432); R-(+)-7-Chloro-8-hydroxy-1-phenyl-2,3,4,5-tetrahydro-1H-3-benzazepine (SCH23390 hydrochloride); 2′, 5′-Dideoxy-3′-ATP; 4′,5,7-Trihydroxyisoflavone; 5,7-Dihydroxy-3-(4-hydroxyphenyl)-4H-1-benzopyran-4-one (genistein); TrkB/Fc Chimera human; (R)-N4-Hydroxy-N1-[(S)-2-(1H-indol-3-yl)-1-methylcarbamoyl-ethyl]-2-isobutyl succinamide (gallardin); Diethyldithiocarbamic acid (DETC); 7, 8-dihydroxyflavone hydrate; and BDNF. (2) Tocris Bioscience (Ellisville, MO, USA):1-(2-Diphenylmethoxyethyl)-4-(3-phenyl-2-propenyl)-piperazine dihydrochloride (GBR12783 dihydrochloride), (±)-1-Phenyl-2, 3, 4, 5-tetrahydro-(1H)-3-benzazepine-7, 8-diol hydrobromide (SKF38393 hydrobromide); (9R,10S,12S)-2,3,9,10,11,12-Hexahydro-10-hydroxy-9-methyl-1-oxo-9,12-epoxy-1H-diindolo[1,2,3-fg:3′,2′,1′-kl]pyrrolo[3,4-i][1], [6]benzodiazocine-10-carboxylic acid, hexyl ester (KT5720); and 1,4-Diamino-2,3-dicyano-1,4-bis[2-a minophenylthio]butadiene (U0126). All drugs were made as concentrated stock solutions in dH2O, except picrotoxin, genistein, KT5720, U0126, gallardin and 7, 8-dihydroxyflavone, which were made as stocks in 100% DMSO. The final concentration of DMSO was no more than 0.1% on final application. Drugs were applied in the ACSF using a continuous gravity fed bath application unless specifically stated.

### Statistics

All data are expressed as the mean ± SEM. The amplitudes of the EPSPs were normalized to the average baseline EPSP amplitude. All statistical tests were conducted using Excel 2007 or Graphpad Prism 4.0. Unless specified, the P value indicated for comparisons of the amplitude of LTP are determined using student's *t* test on data obtained at 30-min post HFS. A two-way ANOVA was performed to test any effect of treatment and time on the access resistance or the membrane input resistance. A P<0.05 was considered statistically significant for all cases.

### Dual-immunofluorescence experiments

Immunofluorescence experiments were performed as previously described [66]. Fluorescent immunohistochemistry was performed using the following primary antibodies: rabbit monoclonal anti-MMP17 (1∶100, ab51075, Abcam, Cambridge, MA), rabbit monoclonal anti-MMP16 (1∶100, ab73877, Abcam, Cambridge, MA), mouse monoclonal anti-CaMKIIα (1∶1000, 05-532, Millipore, Lake Placid, NY), and rabbit polyclonal anti-TrkB antibody (1∶1000, sc-12, Santa Cruz Biotechnology, Santa Cruz, CA). Co-localization of MMP17 with CaMKIIα and TrkB with CaMKIIα was determined by dual-immunofluorescent labeling. Representative BLA sections (from Bregma -2.30 mm to -3.14 mm) were rinsed 3x (10 min each) in PBS, permeabilized with 0.5% Triton-X 100 in PBS, and incubated for 48 hrs at 4°C with two appropriate primary antibodies diluted in 0.5% Triton-X/PBS solution. Sections were rinsed 3x (10 min each) in PBS and then incubated at room temperature for 2 hrs with specific Alexa-Fluor secondary antibodies (1∶500, Molecular Probes, Invitrogen, and Carlsbad, CA, USA), Alexa-Fluor 488 goat anti-mouse IgG and Alexa-Fluor 568 goat anti-rabbit IgG. Following incubation with secondary antibodies, sections were rinsed 3x (10 min each) in PBS and 1x in 0.05 M phosphate buffer (PB), mounted on gelatin-coated glass slides and coverslipped using Vectashield fluorescence mounting medium (Vector Laboratories, Inc., Burlingame, CA). Confocal spinning disk laser microscopy was used to analyze dual-immunofluorescence and to obtain high-resolution photomicrographs using an Orca R2 cooled CCD camera (Hammamatsu, Bridgewater, NJ) mounted on a Leica DM5500B microscope (Leica Mircosystems, Bannockburn, IL) equipped with a CSU10B Spinning Disk (Yokagawa Electronic Corporation, Tokyo, Japan).

### Antibody specificity

The antibody against MMP-17 was developed from a synthetic peptide corresponding to residues on human MMP-17. MMP-17 antibody specificity was confirmed with Western blots by detection of a single band of approximately 58 kDa. MMP-16 antibody was developed against the synthetic peptide mapping at the C-terminal of human MMP16. Specificity of the antibody was confirmed with Western blots by detection of the single band at 57 kDa. The TrkB antibody was raised against a peptide mapping within the C-terminal cytoplasmic domain of Trk B of mouse origin. Specificity of the antibody was confirmed by the manufacturer (Santa Cruz Biotechnology) by detecting single band at 145 kDa, and has been confirmed previously [67]. The CaMKIIα antibody was developed against a synthetic peptide corresponding to the N-terminus of rat CaMKIIα and its specificity has been described previously [68].

### Single Cell RT-PCR

Following recording, cell cytoplasm was aspirated into the recording pipette and expelled into a microcentrifuge tube. RT-PCR reactions were performed for testing mRNA expression using cDNA transcribed from individual cells. The RT product was then amplified in two cycles, the first amplification step using V3 (dT)_24_ primer and cycling conditions of 95°C for 3 min, 50°C for 2 min and 72°C for 3 min. For the second amplification step using V1 (dT) _24_ primer the cycling conditions were the same as mentioned above with 20-cycle PCR amplification and a 6s extension per cycle. The amplified cDNA from each cell was screened for the expression of 18S rRNA as a positive control marker. The amplified cDNA was subjected to another amplification step using 2 µl of cDNA from each cell as a template and 100 nM of each of the primers for D_1_, D_5_, TrkB, MMP17, ERK_1/2_ and CamKIIα. PCR was performed using a 10 min hot start at 95°C followed by a 40 cycle program (94°C for 40 sec, 56°C for 40 sec and 72°C for 1 min).
